# Collection of Monoclonal Antibodies Targeting SARS-CoV-2 Proteins

**DOI:** 10.3390/v14020443

**Published:** 2022-02-21

**Authors:** Marina Pribanić Matešić, Paola Kučan Brlić, Tihana Lenac Roviš, Željka Mačak Šafranko, Abigael Eva Chaouat, Karmela Miklić, Suzana Malić, Nina Ivanković, Maren Schubert, Federico Bertoglio, Alemka Markotić, Ofer Mandelboim, Stipan Jonjić, Ilija Brizić

**Affiliations:** 1 Center for Proteomics, Faculty of Medicine, University of Rijeka, Braće Branchetta 20, 51000 Rijeka, Croatia; marina.pribanic.matesic@medri.uniri.hr (M.P.M.); paola.kucan@medri.uniri.hr (P.K.B.); tihana.lenac@medri.uniri.hr (T.L.R.); karmela.miklic@medri.uniri.hr (K.M.); suzana.malic@medri.uniri.hr (S.M.); 2 Research Department, University Hospital for Infectious Diseases “Dr. Fran Mihaljević”, Mirogojska 8, 10000 Zagreb, Croatia; zmacak@bfm.hr (Ž.M.Š.); nina_ivankovic@hotmail.com (N.I.); alemka.markotic@bfm.hr (A.M.); 3 The Concern Foundation Laboratories at the Lautenberg Center for Immunology and Cancer Research, The Hebrew University Hadassah Medical School, Jerusalem 9112001, Israel; bigou@walla.com (A.E.C.); oferm@ekmd.huji.ac.il (O.M.); 4 Institut für Biochemie, Biotechnologie und Bioinformatik, Abteilung Biotechnologie, Technische Universität Braunschweig, Spielmannstr. 7, 38106 Braunschweig, Germany; maren.schubert@tu-braunschweig.de (M.S.); f.bertoglio@tu-braunschweig.de (F.B.)

**Keywords:** SARS-CoV-2, monoclonal antibodies, variants of concern, COVID-19

## Abstract

In early 2020, the COVID-19 pandemic sparked a global crisis that continues to pose a serious threat to human health and the economy. Further advancement in research is necessary and requires the availability of quality molecular tools, including monoclonal antibodies. Here, we present the development and characterization of a collection of over 40 new monoclonal antibodies directed against different SARS-CoV-2 proteins. Recombinant SARS-CoV-2 proteins were expressed, purified, and used as immunogens. Upon development of specific hybridomas, the obtained monoclonal antibody (mAb) clones were tested for binding to recombinant proteins and infected cells. We generated mAbs against structural proteins, the Spike and Nucleocapsid protein, several non-structural proteins (nsp1, nsp7, nsp8, nsp9, nsp10, nsp16) and accessory factors (ORF3a, ORF9b) applicable in flow cytometry, immunofluorescence, or Western blot. Our collection of mAbs provides a set of novel, highly specific tools that will allow a comprehensive analysis of the viral proteome, which will allow further understanding of SARS-CoV-2 pathogenesis and the design of therapeutic strategies.

## 1. Introduction

In December of 2019, an outbreak of a previously unknown coronavirus began in Wuhan, China. Severe respiratory illness, COVID-19, has spread rapidly throughout the world, and the World Health Organization declared a pandemic of this novel virus, severe acute respiratory syndrome coronavirus 2 (SARS-CoV-2), on 11 March 2020. As of 29 December 2021, 281,808,270 cases had been confirmed, including 5,411,759 lethal outcomes (WHO COVID-19 Dashboard). SARS-CoV-2 is a member of the family Coronaviridae, and shares significant sequence similarity with two other pathogenic coronaviruses, SARS-CoV and Middle East respiratory syndrome CoV (MERS-CoV), causative agents of severe respiratory infections which emerged in 2002 and 2012, respectively [1,2]. SARS-CoV-2 easily spreads and infects individuals of all ages, causing different clinical conditions, from asymptomatic or mild disease to severe respiratory manifestations which require hospitalization [3]. Its genome is a positive-sense, single-stranded RNA of ~30 kb in length which encodes 16 non-structural proteins, four structural proteins (Nucleocapsid, Spike, Envelope, and Membrane) and several accessory factors [4,5]. With the onset of the pandemic, great effort was invested into research of this novel coronavirus, resulting in the rapid development of reagents applied in research, diagnostics, and treatment, including several vaccines. Despite the progress made, many questions regarding the involvement of viral proteins in the biology and pathogenesis of SARS-CoV-2 are still open, and further development of research tools, such as monoclonal antibodies (mAbs), is of the utmost importance. SARS-CoV-2, like other RNA viruses, is prone to the development of mutations while adapting to the human host, and multiple variants have already emerged [6]. Therefore, monoclonal antibodies that are able to recognize proteins of different SARS-CoV-2 variants have an additional advantage. Furthermore, antibodies for most non-structural proteins and accessory factors are still either unavailable or characterized exclusively on recombinant proteins or transfected cells, which potentially misrepresents their performance on infected cells. Here we report the development and characterization of over 40 SARS-CoV-2 monoclonal antibodies directed against structural proteins (Nucleocapsid and Spike), several non-structural proteins (nsp1, nsp7, nsp8, nsp9, nsp10, nsp16) and accessory factors (ORF3a, ORF9b). Our work provides a collection of monoclonal antibodies against different SARS-CoV-2 proteins that will allow a comprehensive analysis of viral proteins and provide tools to systematically investigate viral pathogenesis in the future studies.

## 2. Materials and Methods

### 2.1. Production and Purification of SARS-CoV-2 Recombinant Proteins and Antibodies

Sequences of Nucleocapsid protein (N) and non-structural proteins were obtained from GenBank (severe acute respiratory syndrome coronavirus 2 isolate Wuhan-Hu-1, NC_045512.2), codon-optimized for bacterial expression by Genscript using OptimumGeneTM algorithm and subcloned into pET22b+ vector. The 6-His tag was added either C- or N-terminally. Proteins were expressed using Escherichia coli BL21 pREP4 cells. Bacteria carrying expression vectors encoding SARS-CoV-2 proteins were grown overnight in 25 mL of LB medium and inoculated into 500 mL LB medium with appropriate antibiotics (100 µg/mL of ampicillin, 50 µg/mL of kanamycin) and incubated at 37 °C. When appropriate optical density was reached (OD600 = 0.6 − 0.8), expression of recombinant protein was induced using isopropyl-β-D-thiogalactopyranoside (IPTG). Bacterial cultures were then incubated for 3 h at 30 °C, or 16 h at 16 °C. One milliliter of bacterial suspension was collected, cells were lysed, and protein production was confirmed by Coomassie staining and Western blot (WB) using anti-His peroxidase (POD) as a secondary antibody. The remaining bacterial cells were harvested and lysed using GLB lysis buffer (6 M guanidinium hydrochloride, 20 mM sodium phosphate, 500 mM sodium chloride, pH 7.8, with added Roche complete protease inhibitor, EDTA free, 0.5% Tween-20 and 15 mM β-mercaptoethanol) and sonication. Proteins were purified from the lysate on a HisTrap Nickel column (Cytiva, Sweden) using Äkta Prime. Eluted fractions were analyzed using Coomassie staining and WB. Proteins were concentrated in phosphate buffered saline (PBS), pH 7.4, using a Millipore Amicon Ultra-15 centrifugal filter, and analyzed. The His-tagged Wuhan-Hu-1 Spike Glycoprotein Receptor Binding Domain (RBD), was expressed using a pCAGGS expression vector as described in [7]. In brief, HEK293T cells were grown in Roswell Park Memorial Institute (RPMI) 1640 media (PAN-Biotech GmbH) supplemented with the FBS standard (PAN-Biotech GmbH) (10%), MEM NEAA (100×) (PAN-Biotech GmbH, Aidenbach, Germany), and sodium pyruvate (PAN-Biotech GmbH) (final concentration 0.1 mM). Cells were seeded 24 h before transfection to achieve an 80% confluency the next day. For each flask layer, the transfection mixture was prepared by mixing 19 μg of purified plasmid, 185 uL of polyethylenimine (PEI) solution (1 mg/mL), and 2.8 mL of Dulbecco’s modified Eagle’s media (PAN-Biotech GmbH) for 20–30 min at RT. Next, the flask media were removed, and the transfection mixture was added. After incubating for 2 min, the media were returned to the culture flask and the flask was placed back in the incubator. After 24 h, the media were exchanged with the HyClone HyCell TransFx-H Medium (Cytiva, previously GE Healthcare, Chicago, IL, USA) supplemented with MEM NEAA (100×) (PAN-Biotech GmbH), sodium pyruvate (PAN-Biotech GmbH) (final concentration: 0.1 mM), penicillin–streptomycin (PAN-Biotech GmbH) (final concentrations: penicillin 10 U/mL; streptomycin10 μg/mL), and L-Glutamine (PAN-Biotech GmbH) (final concentration: 0.2 mM). Media were collected and replaced every 3–5 days for 1–2 weeks. The recombinant His proteins were purified from the supernatant using an ÄKTA PureLiquid Chromatography System (GE Healthcare/Cytiva) equipped with HisTrap HP columns packed with Ni Sepharose affinity resin. For antibody production, hybridomas were maintained in RPMI 1640 supplemented with 10% of fetal bovine serum. When cells were 80–90% confluent, the medium was replaced with ISF-1 (Sigma-Aldrich, St. Louis, MO, USA) medium without serum. Antibody containing supernatant (serum-free) was collected after 5 to 7 days and diluted in binding buffer (20 mM phosphate buffer, pH 7.0) in ratio 1:1, purified on HiTrap Protein G HP column and eluted with 0.1 M glycine, pH 2.7. Äkta Pure was used for antibody purification. A short peptide sequence within the N-terminal region of ORF3a, amino acid sequence KQGEIKDATPSDFVR, was ordered as keyhole limpet hemocyanin (KLH)-conjugated synthetic peptide and used as ORF3a immunogen. S1-S2 His-tagged recombinant proteins for wild-type (WT, Wuhan and Spike with trimerization domain) and S1-His variants of concern (B.1.351 with mutations K417N + E484K + N501Y; B.1.617 with mutations L452R + E484Q; B.1.617.2 with mutations L452R + T478K + D614G; B.1.1.529 (Omicron) RBD-His (aa319–541) with mutations G339D, S371L, S373P, S375F, K417N, N440K, G446S, S477N, T478K, E484A, Q493K, G496S, Q498R, N501Y, Y505H) were produced baculovirus-free as described before [8]. Listed mutations in the RBD domain are indicated in Supplementary Table S1. In brief, genes were subcloned into a NcoI/NotI version of the pOpiE2 expression vector. High Five insect cells cultivated in EX-CELL 405 media were transfected at a density of 4 × 10^6^ cells/mL, pipetting 1 µg DNA/10^6^ cells and 4 µg 40 kDa PEI/10^6^ cells subsequently directly into the cell suspension. About 8 h after transfection, cells were diluted to a density of 1 × 10^6^ cells/mL by addition of fresh EX-CELL 405 media and 48 h after transfection the culture volume was doubled. The supernatant was harvested 96 h after transfection in stepwise centrifugation (4 min at 180× *g* followed by 20 min at >3000× *g*). After 0.2 µm filtration and addition of 0.5 M NaCl the His-tagged proteins were purified by a HisTrap excel column followed by SEC (16/600 Superdex 200 kDa pg) on Äkta systems according to the manufacturer protocol.

### 2.2. Immunization and Generation of Hybridomas

Two BALB/c mice per immunogen were immunized with purified antigen, using 50 µg per mouse, in Freund’s adjuvant and two weeks later mice were boosted with the same protein. Blood was collected from the tail vein after 10–15 days after the second immunization and serum was subjected to enzyme-linked immunosorbent assay (ELISA) to check antibody titer. Serum from non-immunized mouse was used as a control. Mice were then boosted with the same immunogen, euthanized after 3–5 days and splenocytes were subjected to fusion with SP2/O myeloma cells (ratio 1:1). Cells were cultivated in 96-well plates in 20% RPMI 1640 medium with hypoxanthine, aminopterin, and thymidine (HAT) used for hybridoma selection. Hybridoma cultures were screened for the production of antibodies using ELISA. Positive hybridomas were cloned. The Animal Welfare Committee at the University of Rijeka, Faculty of Medicine and National ethics committee approved all animal experiments (525-10/0543-20-3).

### 2.3. Enzyme-Linked Immunosorbent Assay (ELISA)

ELISA was used for screening of induction of antibody responses in sera of immunized mice, for selection of antigen-specific hybridoma cell lines and mAb isotype determinations. In brief, 2–5 µg/mL of target protein was coated on high-binding ELISA plates (MICROLON^®^ High Binding, Greiner Bio-One, Kremsmünster, Austria) in carbonate/bicarbonate coating buffer pH 9.6. Plates were incubated overnight at 2–8 °C, washed two times with tap water [9], and saturated with blocking buffer (2.5 g bovine serum albumin (BSA) in 1000 mL demineralized water with 50 µL Tween-20 and 5 mL 10% azide) for 2 h at room temperature. Samples were added in PFT buffer (PBS containing 1% FCS and 0.05% Tween 20) and were diluted depending on the assay: serum samples serially diluted from 1:50 to 1:102400; hybridoma and clone cell supernatants diluted 1:2 or undiluted. After 1–2 h sample incubation, plates were washed with tap water and incubated for 1 h with Peroxidase AffiniPure Goat Anti-Mouse IgG (H + L) antibody (Jackson ImmunoResearch, West Grove, PA, USA, 115-035-003) diluted 1:1000 in PFT. After washing six times, a colorimetric reaction was performed with a 0.6 mg/mL solution of o-phenylenediamine dihydrochloride (OPD) (Sigma, P8412) in citrate buffer pH 5.5 and 0.001% of 30% hydrogen peroxide at RT for 5–10 min. After stopping the reaction with 1 M sulfuric acid, the absorbance was measured using a TriStar LB 941 multimode microplate reader with the wavelength set at 490 nm and the reference filter set at 630 nm. In all ELISAs, adequate negative controls were used, i.e., sera from unimmunized mice, isotype control matched irrelevant antibody, or use of plates coated with irrelevant protein. ELISA data were analyzed using Graphpad Prism software (version 8.1.0).

### 2.4. Viruses

SARS-CoV-2 viruses used for infection of ACE-2 transfected HEK 293T cells were USA-WA1/2020 (NR-52281, here named as WT1), obtained from BEI resources; B.1.1.7 (Alpha) and B.1.617.2 (Delta) strains, isolated from oropharyngeal swabs and B.1.351 (Beta), kindly provided by Dr. Alex Sigal (Nelson R Mandela School of Medicine, UKZN). SARS-CoV-2 virus used for infection of Vero E6 cells was a SARS-CoV-2/297/20 Zagreb (here named as WT2), isolate derived from a positively tested nasopharyngeal swab in Zagreb, Croatia (GISAID database ID: EPI_ISL_451934) passage 5 [10,11] or 0707*149 (B.1.617.2, Delta) passage 3 isolated from a nasopharyngeal swab. All virus stocks were propagated (four passages) and tittered on Vero E6 cells. All infection and cell culture processing experiments were performed in a biosafety level 3 (BSL-3) facility.

### 2.5. Flow Cytometry and Immunofluorescence

293T-ACE2 cells [12] were infected with the different SARS-CoV-2 strains at an MOI of 0.5. After 24 h, cells were harvested and stained with the primary anti-SARS-CoV-2 antibody at 4 °C for 1 h, cells were washed in FACS buffer (1% BSA and 0.05% Sodium Azide in phosphate-buffered saline) and secondary antibody was added for 30 min at 4 °C. Then, cells were washed in FACS buffer and fixed with 4% paraformaldehyde for 20 min followed by flow cytometry. The following secondary antibody was used: Alexa Fluor 647-conjugated Goat Anti-Mouse IgG (Cat# 115-606-062, Jackson ImmunoResearch Laboratories). Vero E6 cells were infected with MOI of 0.2, and incubated for 24 h. Cells were fixed with 2% PFA for 1 h, washed in PBS, pH = 7,3), permeabilized with permeabilization buffer (Cat# 00-8333-56, Invitrogen) and stained with SARS-CoV-2 antibody supernatants diluted in permeabilization buffer 1:1, for 1 h at room temperature, followed by secondary Rat-Anti-Mouse IgG1 FITC antibody (Cat# 11-4015-80, Invitrogen) for 30 min at room temperature. All flow cytometry data were analyzed using FlowJo_v10 (Tree Star) software. For immunofluorescence, cells were seeded at a density of 25,000 cells per well in 96 well plates with a transparent bottom and infected with the virus at an MOI of 0.2. After 24 h of incubation, cells were fixed with 2% paraformaldehyde (PFA). Cells were washed with phosphate buffered saline (PBS) and permeabilized with methanol for 5 min. Methanol was washed 2× with PBS and incubated with mAbs for 1 h at room temperature. mAbs were used as hybridoma cell culture supernatants diluted in PBS (1:2) or, if purified, by diluting the original 1 mg/mL antibody stocks 1:100 in PBS (clones S1.01, N.01, and N.09). Mock infected cells and matching isotype controls were used as a control. The samples were stained for 40 min with secondary antibodies coupled to TRITC fluorophores (Sigma, SAB3701100), stained with DAPI for 5 min, washed with PBS and mounted using Mowiol mounting medium and analyzed at room temperature with a Leica TCS SP8 confocal laser scanning microscope using an HC PL APO 40×/1.30 OIL CS2 objective and LasX acquisition software (version 3.5.6.21594) without gamma adjustments. At least three independent experiments were performed for each subject of analysis.

### 2.6. Western Blot

Western blot was used to detect recombinant and proteins in lysates of infected cells. Recombinant proteins (2–4 µg) or infected and mock-infected cell lysates (48 h, MOI 0.5) were subjected to gel electrophoresis before transfer to nitrocellulose membrane for 1 h at 400 mA. Cells were lysed in radioimmunoprecipitation assay (RIPA) buffer containing protease inhibitors (Roche Applied Science, Penzberg, Germany). The PageRuler Prestained protein ladder (ThermoFisher, Waltham, MA, USA) was used for determination of protein molecular weight. Membranes were blocked with 5% milk in 1× TBS-T. Purified monoclonal antibodies (clones N1.01 and N1.09; original stock concentration 1 mg/mL) were diluted 1:250 to 1:500 and antibody containing supernatants were diluted 1:1 in blocking solution, followed by overnight incubation with shaking at 4 °C. Membranes were washed three times with TBS-T for 10 min each and incubated for 1 h at room temperature with anti-mouse Fab secondary antibody (anti-mouse Fab2 POD, Jackson 115-036-072) in blocking solution or anti-His POD secondary antibody for detection of His-tagged recombinant proteins. After incubation, membranes were washed three times with TBS-T for 10 min each and placed in a developing folder. The developing reagent (ECL, GE Healthcare) was added to a membrane and image was obtained using ImageQuant Las 4000.

## 3. Results

**Figure 1:**
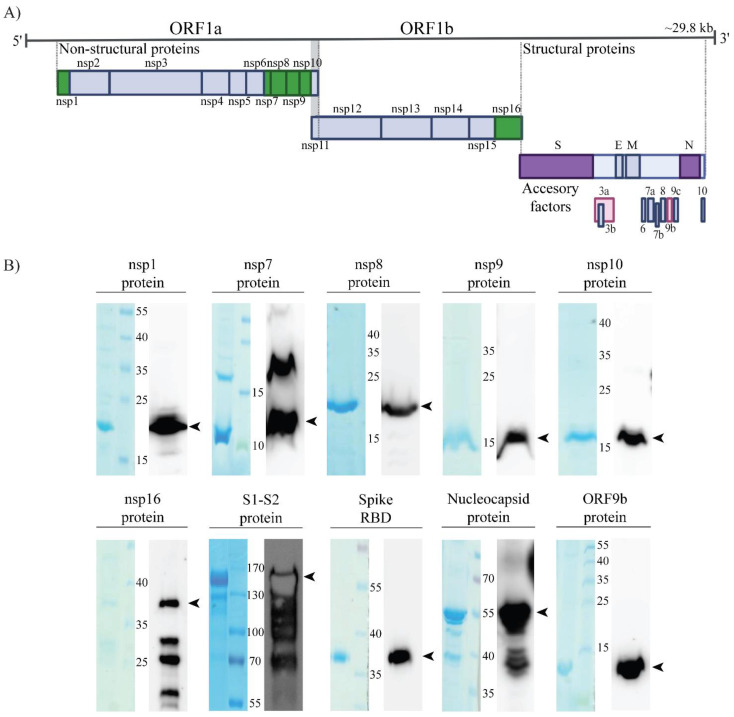
Expression of SARS-CoV-2 recombinant proteins. (**A**) Graphical representation of SARS- CoV-2 viral genome. Immunogens generated in the study are shown as colored boxes: non-structural proteins (nsp1, nsp7, nsp8, nsp9, nsp10, nsp16) are shown as green boxes, Spike and Nucleocapsid protein as purple, and accessory factors ORF3a and ORF9b as pink boxes. (**B**) Analysis of expressed and purified His-tagged immunogens by SDS-PAGE (left) and Western blotting (right) with anti-His POD antibody. SDS-PAGE gel was stained with Coomassie blue. Lane shows purified protein, molecular mass markers (in kDa) are indicated in the middle. The arrowheads indicate bands near expected molecular weights.

To generate the majority of SARS-CoV-2 proteins for immunization, we expressed respective coding regions as full-length proteins with either N- or C-terminal His tag in E. coli using pET-22b(+) expression vector (Figure 1, Supplementary Table S2). Immunogen for wild-type Wuhan Spike protein was expressed either as RBD His-tagged domain using pCAGGS expression vector in HEK293T cells [7] or as 6x-His-tagged S1-S2 domain baculovirus-free produced in High Five cells [13]. For the generation of ORF3a antibodies, KLH-conjugated synthetic peptide encompassing a short peptide sequence within the N-terminal region was used as an immunogen. All in-house generated proteins were tested for size and purity by SDS-PAGE followed by Coomassie blue staining and by Western blot analysis with anti-His POD antibody (Figure 1B). The analysis of recombinant proteins showed, for most of the generated proteins, protein bands corresponding to expected molecular weights (Supplementary Table S2). For some proteins, multiple bands were detected, e.g., nsp16 was detected on the predicted size of 35–40 kDa (arrowhead Figure 1B), with several additional bands of lower molecular weight, as observed by others [14]. Similarly, additional bands of lower molecular weight observed for the Nucleocapsid protein are also reported in publications [15,16] or datasheets of other antibody producers (Proteintech cat.no. 1B3C3, Cell Signaling cat.no. 33717). Recombinant nsp7 protein was detected of the expected size (10 kDa) and of higher molecular weight, probably due to its reported dimerization [17]. Analysis of antibodies raised against ORF3a showed binding to the ORF3a peptide conjugated to the bovine serum albumin (BSA) carrier, and thus, protein band corresponding to the molecular weight of ORF3a could not be detected. The presence of multiple bands for recombinant Spike protein, corresponding to different forms of the protein, has been reported previously [18].

In summary, we obtained immunogens for 10 distinct viral proteins for immunization.

Following immunization of mice and fusion of splenocytes with the myeloma cell line, we selected and cloned several hybridomas for each immunogen. In total, more than 8000 supernatants were tested for antibody binding on the respective immunogen-coated ELISA plates. A cross-reactivity test was performed using His-tagged control protein (not shown). At least one clone from each parental hybridoma specifically recognizing a respective antigen in ELISA was chosen for further characterization (Table 1).

**Figure 2:**
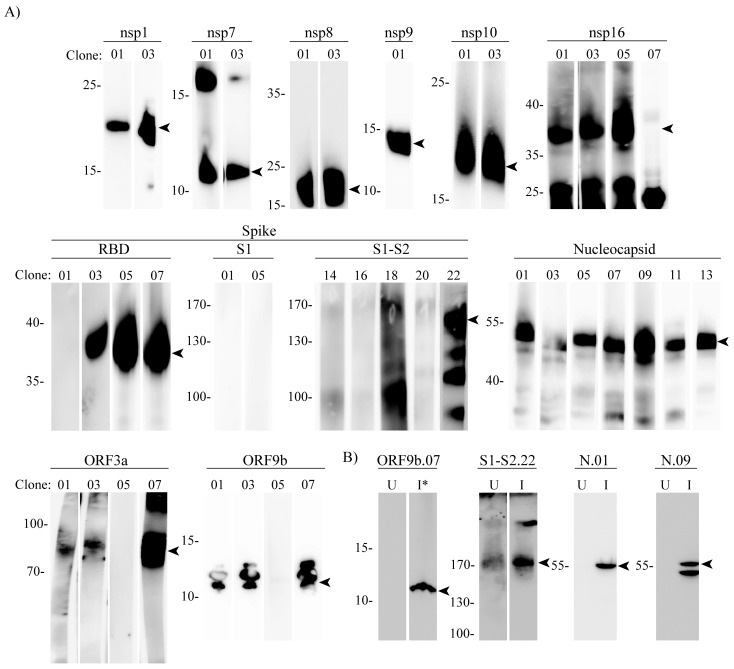
Validation of SARS-CoV-2 specific antibodies by Western blot analysis. (**A**) Recombinant proteins or KLH-conjugated peptides (ORF3a) were subjected to gel electrophoresis and detected using SARS-CoV-2 mAbs raised against indicated immunogens. Molecular mass markers (in kDa) are indicated on the left. (**B**) Lysates of uninfected (U) or, WT2 infected (I) or Delta infected (I*) cells were prepared at 48 h post infection and analyzed by Western blotting using specific mAbs raised against the indicated proteins. Molecular mass markers (in kDa) are indicated on the right. Arrowheads indicate bands near expected molecular weights.

Next, we subjected the recombinant immunogen proteins to denaturing SDS-PAGE and analyzed them by immunoblotting using hybridoma supernatants selected in the previous step. For most of the tested supernatants we could observe specific band(s) corresponding to the immunogen, confirming their recognition of denatured protein (Figure 2A).

Given that the recognition of a recombinant protein does not guarantee that the antibody will recognize the endogenous antigen, using the new set of generated antibodies we validated some of the mAbs by WB analysis of infected Vero E6 cell lysates (Figure 2B). All validated mAbs detected proteins exclusively in infected, but not in uninfected, cell lysates and the migration of proteins in SDS-PAGE gels correlated well with the predicted molecular weight of the proteins

**Figure 3:**
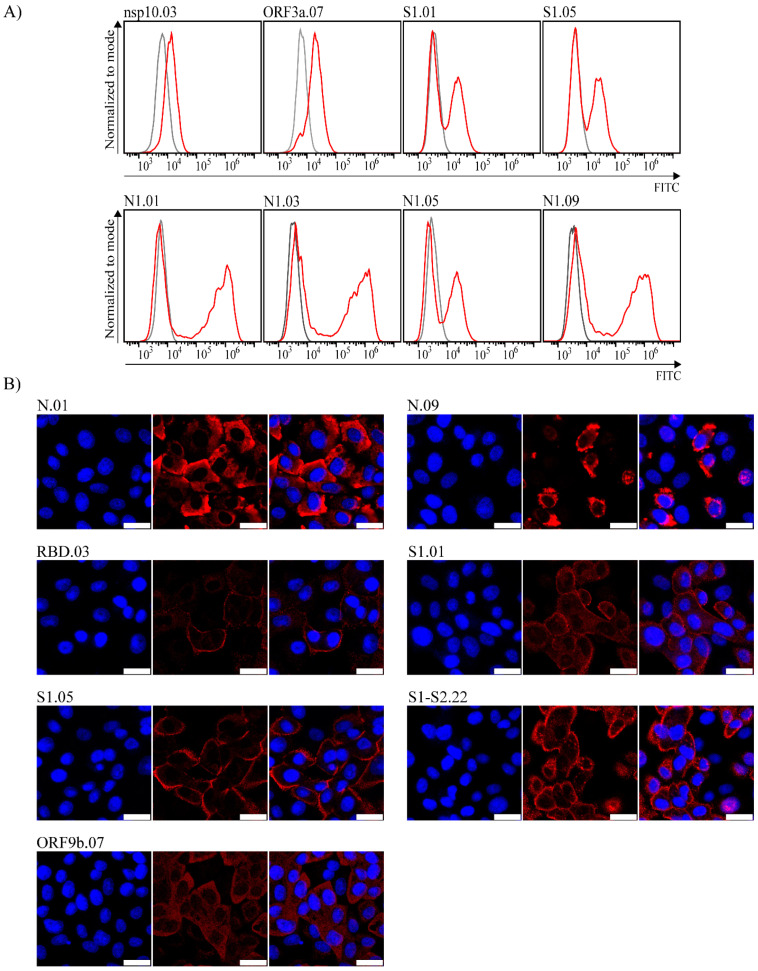
Detection of SARS-CoV-2 proteins in infected Vero E6 cells 24 h p.i. by flow cytometry or confocal microscopy. (**A**) Infected cells were analyzed by intracellular staining and flow cytometry with respective monoclonal antibodies following secondary FITC-coupled antibodies (red histogram). Mock infected cells were used as negative control (grey histogram). (**B**) Infected cells were stained by respective monoclonal antibodies following secondary TRITC-coupled antibodies (red) and nuclei staining by DAPI (blue). Images were obtained using Leica confocal microscope. The size bar (white line) corresponds to 25 µm. Representative mAb clones are shown and are indicated above the panel.

Furthermore, to investigate whether generated mAbs are able to detect the native SARS-CoV-2 proteins in infected cells, we infected Vero E6 cells with SARS-CoV-2/297/20 Zagreb and tested the mAbs by flow cytometry and confocal microscopy (Figure 3). At 24 h post-infection, using intracellular staining of infected cells, we identified several anti-SARS-CoV-2 mAbs able to recognize viral proteins by flow cytometry. Clear staining was observed for the majority of anti-Nucleocapsid antibodies and several anti-Spike antibodies (Figure 3A), confirming their performance against the native antigen. Importantly, our mAbs targeting nsp10 and ORF3a detected SARS-CoV-2 infected cells by flow cytometry. To our knowledge, this has not been reported for commercially available nsp10 and ORF3a antibodies. Similarly, by using confocal microscopy, we observed a clear signal by several mAbs detecting Nucleocapsid protein, several clones detecting Spike protein, and a single clone detecting ORF9b protein (Figure 3B). As expected, anti-Spike clones showed predominant membrane localization of the Spike protein, while Nucleocapsid protein and ORF9b were detected in the cytoplasm. All clones were tested on uninfected cells as control and showed no unspecific binding (Supplementary Figure S2).

**Figure 4:**
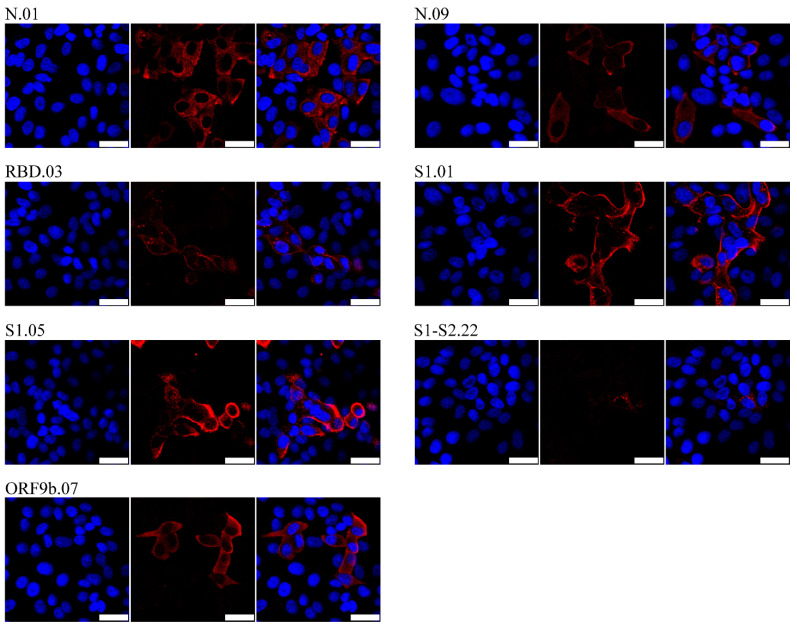
Detection of SARS-CoV-2 proteins in Delta SARS-CoV-2 infected cells by confocal microscopy. SARS-CoV-2 Delta infected Vero E6 cells were analyzed at 24 h p.i. Infected cells were stained by respective monoclonal antibodies following secondary TRITC-coupled antibodies (red) and nuclei staining by DAPI (blue). Images were obtained using a Leica confocal microscope. The size bar (white line) corresponds to 25 µm. Representative mAb clones are shown and indicated above the panel.

Since numerous variants of the SARS-CoV-2 have emerged throughout the world, we determined if our antibodies also recognize variant proteins and could therefore be more widely used. To that aim, Vero E6 cells were infected with Delta variant (0707*149), and 24 h post infection antibodies were tested by confocal microscopy (Figure 4). Again, most of the tested clones, except S1-S2.22 that gave weaker signal, recognized respective proteins in Delta SARS-CoV-2 infected cells suggesting they could be used to detect infection with different virus variants.

**Figure 5:**
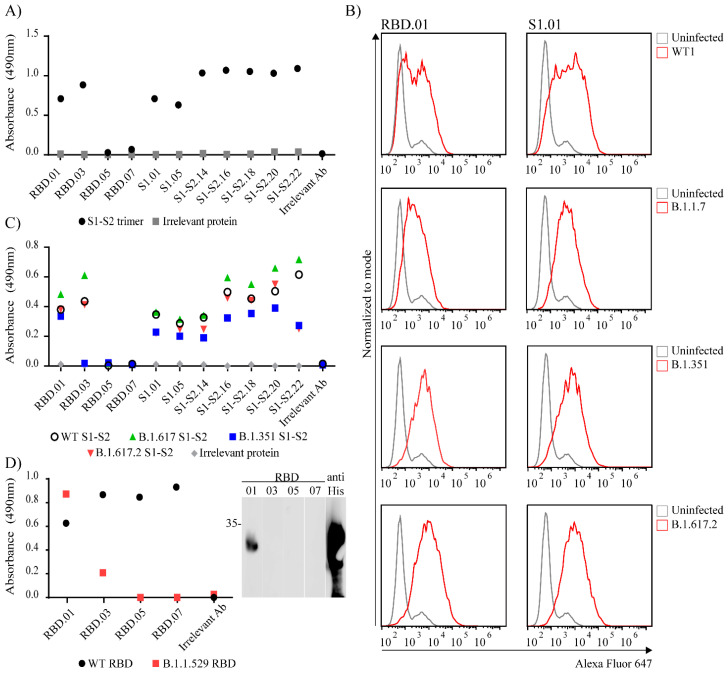
Testing specificity of Spike antibodies. (**A**) All anti-Spike clones were tested in ELISA for their recognition of trimeric Spike protein. (**B**) 293T-ACE2 cells were infected with the WT1 or different SARS-CoV-2 variants and after 24 h stained with indicated anti-Spike clones followed by Alexa Fluor 647-conjugated secondary antibodies (red line). Uninfected cells were used as negative control (grey line). (**C**) Anti-Spike clones generated using full Spike protein as immunogen were tested in ELISA for their specificity against mutated His-tagged Spike protein corresponding to different virus variants. (**D**) Anti-Spike clones generated using RBD protein as immunogen were tested in ELISA (left) and WB (right) for their specificity against mutated His-tagged RBD protein corresponding to B.1.1.529 variant. (**A**,**C**,**D**) For ELISA His-tagged Nucleocapsid protein (grey dot) was coated as negative control and anti-Nucleocapsid antibody (clone N.01) was used as irrelevant antibody. (**D**) For WB RBD B.1.1.529 protein was stained with anti-His POD.

Given that the Spike protein is the most important vaccine target and has huge interest of the scientific community, we decided to further characterize anti-Spike antibodies (Supplementary Table S3). First, we determined if the antibodies recognize Spike in a trimeric form. To that aim, we tested anti-Spike clones listed in Table 1 on ELISA plates coated with recombinant WT Spike protein with the trimerization T4 domain preserved. As seen in Figure 5A, most of the anti-Spike clones were able to bind to trimeric Spike protein suggesting they can recognize epitopes presented in the correct conformation of the intact S protein trimer. Next, we wanted to determine if anti-Spike mAbs recognize Spike proteins of different variants of concern. To that aim, we infected ACE-2 transfected HEK293T cells with either USA-WA1/2020 (NR-52281; WT), B.1.1.7, B.1.617.2 or B.1.351 SARS-CoV-2 virus. Twenty-four hours post infection we performed surface staining of cells with anti-Spike clones RBD.01 and S1.01 and analyzed them by flow cytometry (Figure 5B). Both RBD.01 and S1.01 antibodies nicely distinguished infected cells in the case of all tested variants. Anti-Spike antibodies listed in Table 1 were also tested by ELISA for their recognition of recombinant Spike protein of the following variants of concern B.1.351, B.1.617, B.1.617.2 (Figure 5C) and RBD protein of B.1.1.529 variant (Figure 5D). As depicted in Figure 5C, all of the anti-Spike clones that were raised using full wild type Spike protein as immunogen, recognized mutated S1 Spike proteins corresponding to variants B.1.351, B.1.617 and B.1.617.2. Out of four mAbs raised against wt RBD, a single mAb, RBD.01, recognized B.1.1.529 (Omicron) RBD protein in ELISA and Western blot (Figure 5D). Furthermore, five out of seven Spike mAbs raised against full Spike protein are specific for wt RBD protein (Supplementary Figure S1). However, none of these mAbs bound Omicron RBD. Therefore, as expected, mutations in Omicron RBD disrupted the ability of the majority of mAbs to recognize this protein, but still we identified one mAb clone, RBD.01, with outstanding ability to bind RBD of all tested variants of concern.

In summary, our data show the generation and validation of a set of novel mouse monoclonal antibodies against 10 SARS-CoV-2 proteins that can be used in different techniques and represent a new valuable tool for future research of the SARS-CoV-2 pathogenesis.

## 4. Discussion

Antibodies are among the most frequently used reagents in biological research. Here, we report the development and characterization of almost 40 new monoclonal antibodies against 10 SARS-CoV-2 proteins, including structural and non-structural proteins, and accessory factors. This collection covers more than one third of the SARS-CoV-2 proteins and may help foster an improved understanding of their biological and structural characteristics.

The Spike protein mediating viral entry into cells is the most important vaccine target and has been most extensively studied due to its importance in the immune response and pathogenesis. Here we generated several anti-Spike antibodies using different antigens and extensively characterized them. We identified antibodies able to recognize Spike protein with ELISA, immunofluorescence microscopy, flow cytometry and Western blot, providing an outstanding resource for studies of Spike protein. Since it has been suggested that some of the epitopes might be inaccessible or hidden in the trimeric form that assembles on the virion surface [19,20], we determined that our antibodies recognize Spike in a trimeric form and that they can recognize epitopes presented in the correct conformation. Next, given that several SARS-CoV-2 variants with S protein mutations have recently emerged, with many of the mutations altering the conformational state of the protein and impairing neutralization efficiency of naturally or vaccine-induced antibodies [21], we checked whether our anti-Spike antibodies might also have reduced ability to recognize mutated proteins, i.e., cells infected with variant viruses. All of our clones raised using full wt Spike protein as immunogen recognized the tested mutated proteins in ELISA, suggesting they can be used as markers of infection for different SARS-CoV-2 variants. In contrast, antibodies raised using wt RBD varied in their recognition of RBDs of different variants. This is not surprising given the numerous mutations in the RBD domain that have been reported for SARS-CoV-2 variants [22]. Importantly, antibody RBD.01 recognized the RBD domain of Spike protein in all variants tested, including B.1.1.529 (Omicron).

Similar to Spike protein, we generated successfully several monoclonal antibodies able to recognize another major structural protein, Nucleoprotein, with ELISA, immunofluorescence microscopy, flow cytometry and Western blot. In addition, we also developed monoclonal antibodies specific for nsp1, nsp7, nsp8, nsp9, nsp10, nsp16, ORF3a and ORF9b proteins. Furthermore, we identified an anti-ORF9b antibody that recognizes ORF9b protein in SARS-CoV-2-infected cells by both immunofluorescence and Western blot. These properties have not been previously demonstrated for commercially available antibodies that were tested using only recombinant proteins or transfectants. Given the important role of ORF9b in the suppression of host innate immunity by disruption of interferon responses [23,24], we are convinced that this antibody will contribute to its further characterization and understanding. Similarly, our antibody to nsp10, an intriguing protein known to form complex with nsp16 that is involved in methylation of a capped RNA strand and necessary for viral immune evasion [25], is the first anti-nsp10 antibody reported for use in flow cytometry and, in particular, on infected cells. Many other antibodies described here cover SARS-CoV-2 non-structural proteins that have been of great interest due to their important role in the immune response: nsp1 that functions as a virulence factor inhibiting host translation [26,27] and resulting in modulation of host immune functions [28], and nsp8 and nsp9 that interfere with IFN response [29]. These SARS-CoV-2 proteins represent promising antiviral drug targets, indicating the need for their further functional and biochemical studies. In addition, it should be noted that one of the key advantages is that all our antibodies are monoclonal, which means they are homogeneous and consistent, allowing potential structural analysis and other advantages of the monospecificity [30]. An additional advantage is the fact that many of the generated antibodies recognize proteins in SARS-CoV-2 infected cells. Nevertheless, many of these antibodies will require further characterization and will be available to scientific community.

It should be noted that we do not exclude that some other clones might be operative in different applications, but their performance might have been underestimated due to the use of clone supernatants and not purified antibodies of a known concentration. This could also explain the differences in ELISA absorbance values observed between different clones in trimer or mutated protein detection experiments. The performance of the antibodies, particularly in conditions of natural infection, might be also be affected by the levels of endogenous proteins, which are often lower than in recombinant systems, or by the interactions with other proteins that can mask antibody-recognition domains [31]. Since most immunogens were purified under denaturing conditions, it is possible that some of antibodies do not recognize epitopes present in the native protein (i.e., conformational epitopes). This might explain their lack of binding to protein in some techniques such as immunofluorescence or flow cytometry in which the native structure of the protein remains well preserved.

In summary, the current study presents a comprehensive resource of SARS-CoV-2 specific mAbs that may allow detailed analyses of many viral proteins expressed during infection.

## Figures and Tables

**Table 1:** Isotype and performance of selected clones in different techniques.

Protein	Clone	Isotype	Western Blot *	IF	Flow Cytometry
**Nucleocapsid protein**	N.01	κ, IgG1	+ **	+	+
	N.03	λ, IgG1	+	-	+
	N.05	κ, IgG1	+	-	+
	N.07	κ, IgG1	+	-	-
	N.09	λ, IgG1	+ **	+	+
	N.11	κ, IgG1	+	+	+
	N.13	κ, IgG1	+	-	+
**Spike protein**	RBD.01	κ, IgG1	-	-	+
	RBD.03	κ, IgG1	+	+	-
	RBD.05	κ, IgG1	+	-	ND
	RBD.07	κ, IgG1	+	-	ND
	S1.01	κ, IgG1	-	+	+
	S1.05	κ, IgG1	-	+	+
	S1-S2.14	κ, IgG2a	+/-	ND	-
	S1-S2.15	κ, IgG2a	ND	-	ND
	S1-S2.16	κ, IgG2b	-	ND	-
	S1-S2.17	κ, IgG2b	ND	+	ND
	S1-S2.18	κ, IgG2b	+/-	ND	-
	S1-S2.19	κ, IgG2b	ND	+	ND
	S1-S2.20	κ, IgG2b	+/-	ND	-
	S1-S2.21	κ, IgG2b	ND	+	ND
	S1-S2.22	κ, IgG2b	+ **	+	-
**ORF3a**	ORF3a.01	κ, IgG1	+	-	-
	ORF3a.03	κ, IgG1	+	-	-
	ORF3a.05	λ, IgG1	-	-	-
	ORF3a.07	λ, IgG1	+	-	+
**ORF9b**	ORF9b.01	κ, IgG2b	+	-	-
	ORF9b.03	κ, IgG1	+	-	-
	ORF9b.05	κ, IgG1	-	-	-
	ORF9b.07	κ, IgG1	+ **	+	-
**Nsp1**	Nsp1.01	κ, IgG1	+	-	-
	Nsp1.03	κ, IgG1	+	ND	-
**Nsp7**	Nsp7.01	κ, IgG2b	+	-	-
	Nsp7.03	κ, IgG1	+	-	-
**Nsp8**	Nsp8.01	κ, IgG1	+	ND	-
	Nsp8.03	κ, IgG1	+	ND	-
**Nsp9**	Nsp9.01	κ, IgG1	+	ND	-
**Nsp10**	Nsp10.01	κ, IgG1	+	-	-
	Nsp10.03	κ, IgG1	+	-	+
**Nsp16**	Nsp16.01	κ, IgG2a	+	-	-
	Nsp16.03	κ, IgG2a	+	-	-
	Nsp16.05	κ, IgG2a	+	-	-
	Nsp16.07	κ, IgG1	-	-	-

* Western blot performance was tested on recombinant protein. ** Antibodies validated in western blot on lysates of infected cells. + positive, - negative, ND—not determined, IF—immunofluorescence.

## Data Availability

All relevant data are available in the manuscript or within the supplemental data.

## Supplementary Materials

The following supporting information can be downloaded at: https://www.mdpi.com/article/10.3390/v14020443/s1, Figure S1: ELISA test of anti-Spike monoclonal antibodies raised against recombinant full WT S1-S2 protein for binding of WT RBD and mutated RBD protein corresponding to B.1.1.529 (Omicron) variant, Figure S2: Testing anti-SARS-CoV-2 mAbs on mock infected cells by confocal microscopy, Table S1: Sequences of RBD domains of SARS-CoV-2 Spike protein in indicated recombinant proteins, Table S2: Immunogen description and resulting mAb clones, Table S3: Performance of anti-Spike antibodies in different techniques on variant Spike/RBD proteins or variant SARS-CoV-2 infected cells.
