# Supplementary material for: Collection of Monoclonal Antibodies Targeting SARS-CoV-2 Proteins
Source: Viruses. 2022 Feb 21;14(2):443. doi: 10.3390/v14020443 (PMC8875891; doi:10.3390/v14020443)
Supplement: Supplementary file 1 [file viruses-14-00443-s001.zip › viruses-1577243-supplementary.pdf]

Supplementary Data

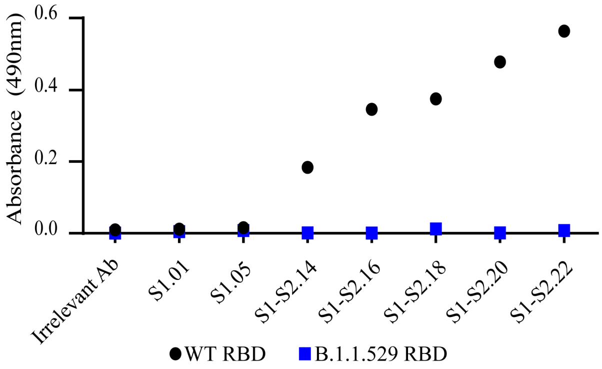

Supplementary Figure S1. ELISA test of anti-Spike monoclonal antibodies raised against recombinant full WT S1-S2 protein for binding of WT RBD and mutated RBD protein corresponding to B.1.1.529 (Omicron) variant. ELISA plates were coated with 2ug/ml of WT- or Omicron-RBD.

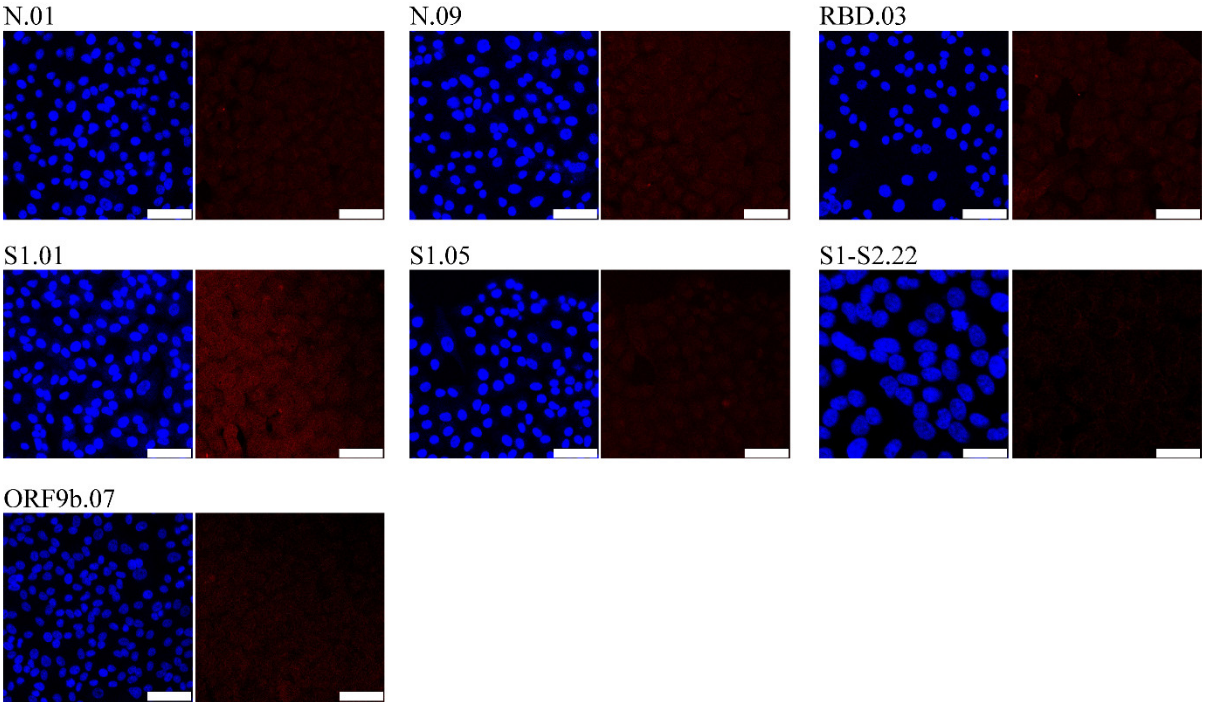

Supplementary Figure S2. Testing anti-SARS-CoV-2 mAbs on mock infected cells by confocal microscopy. Mock infected cells were stained with respective monoclonal antibodies following secondary TRITC-coupled antibodies (red) and nuclei staining by DAPI (blue). Images were obtained using Leica confocal microscope. The size bar (white line) corresponds to 50 μm. Tested mAb clones are indicated above the panel.

Supplementary Table S1: Sequences of RBD domains of SARS-CoV-2 Spike protein in indicated recombinant proteins.

SARS-Cov-2 virus/variant	RBD aminoacid sequence in recombinant protein (mutations are indicated in **bold red**)
Wild-type, Wuhan<br>NCBI ref.seq.<br>YP_009724390.1<br>region 319-541	RVQPTESIVRFPNITNLCPFGEVFNATRFASVYA<br>WNRKRISNCVADYSVLYNSASFSTFKCYGVSPT<br>KLNDLCFTNVYADSFVIRGDEVRQIAPGQTGKI<br>ADYNYKLPDDFTGCVIAWNSNNLDSKVGGNY<br>NYLYRLFRKSNLKPFERDISTEIYQAGSTPCNGV<br>EGFNCYFPLQSYGFQPTNGVGYQPYRVVVLSFE<br>LLHAPATVCGPKKSTNLVKNKCVNF
B.1.351	RVQPTESIVRFPNITNLCPFGEVFNATRFASVYA<br>WNRKRISNCVADYSVLYNSASFSTFKCYGVSPT<br>KLNDLCFTNVYADSFVIRGDEVRQIAPGQTG**N**I<br>ADYNYKLPDDFTGCVIAWNSNNLDSKVGGNY<br>NYLYRLFRKSNLKPFERDISTEIYQAGSTPCNGV<br>**K**GFNCYFPLQSYGFQPT**Y**GVGYQPYRVVVLSFE<br>LLHAPATVCGPKKSTNLVKNKCVNF
B.1.617	RVQPTESIVRFPNITNLCPFGEVFNATRFASVYA<br>WNRKRISNCVADYSVLYNSASFSTFKCYGVSPT<br>KLNDLCFTNVYADSFVIRGDEVRQIAPGQTGKI<br>ADYNYKLPDDFTGCVIAWNSNNLDSKVGGNY<br>NY**R**YRLFRKSNLKPFERDISTEIYQAGSTPCNGV<br>**Q**GFNCYFPLQSYGFQPTNGVGYQPYRVVVLSF<br>ELLHAPATVCGPKKSTNLVKNKCVNF
B.1.617.2	RVQPTESIVRFPNITNLCPFGEVFNATRFASVYA<br>WNRKRISNCVADYSVLYNSASFSTFKCYGVSPT<br>KLNDLCFTNVYADSFVIRGDEVRQIAPGQTGKI<br>ADYNYKLPDDFTGCVIAWNSNNLDSKVGGNY<br>NY**R**YRLFRKSNLKPFERDISTEIYQAGS**K**PCNG<br>VEGFNCYFPLQSYGFQPTNGVGYQPYRVVVLS<br>FELLHAPATVCGPKKSTNLVKNKCVNF
B.1.1.529	RVQPTESIVRFPNITNLCPF**D**EVFNATRFASVYA<br>WNRKRISNCVADYSVLYN**L**A**P**F**F**TFKCYGVSPT<br>KLNDLCFTNVYADSFVIRGDEVRQIAPGQTG**N**I<br>ADYNYKLPDDFTGCVIAWNSN**K**LDSKV**S**GNY<br>NYLYRLFRKSNLKPFERDISTEIYQAG**NK**PCNG<br>V**A**GFNCYFPL**K**SY**S**F**R**PT**Y**GVG**H**QPYRVVVLSF<br>ELLHAPATVCGPKKSTNLVKNKCVNF

Supplementary Table S2: Immunogen description and resulting mAb clones

Protein	Immunogen (range of aminoacid sequence) *	Tag	Vector/expression system	In silico predicted molecular weight **	Resulting mAb clones
Nucleocapsid protein	1 –419	C-term 6x HIS tag	pET22b+, E.coli BL21 pREP4	45,6 kDa	N.01-N.13
RBD	319-541	C-term 6x HIS tag	pCAGGS, HEK293T	25 kDa	RBD.01-RBD.07
S1-S2	14–1208	C-term 6x HIS tag	pOpiE2, High Five insect cells	132 kDa	S1.01, S1.05, S1-S2.14 – S1.S2.22
Nsp1	1-180	C-term 6x HIS tag	pET22b+, E.coli BL21 pREP4	19,8 kDa	Nsp1.01-Nsp1.03
Nsp7	1-83	C-term 6x HIS tag	pET22b+, E.coli BL21 pREP4	9,2 kDa	Nsp7.01-Nsp7.03
Nsp8	1-198	C-term 6x HIS tag	pET22b+, E.coli BL21 pREP4	21,9 kDa	Nsp8.01-Nsp8.03
Nsp9	1-113	N-term 6x HIS tag	pET22b+, E.coli BL21 pREP4	12,4 kDa	Nsp9.01
Nsp10	1-138	N-term 6x HIS tag	pET22b+, E.coli BL21 pREP4	14,8 kDa	Nsp10.01-Nsp10.03
Nsp16	1-298	N-term 6x HIS tag	pET22b+, E.coli BL21 pREP4	33,3 kDa	Nsp16.01-Nsp16.07
ORF9b	1-97	C-term 6x HIS tag	pET22b+, E.coli BL21 pREP4	10,8 kDa	ORF9b.01-ORF9b.07

* NCBI NC_045512.2 was used as reference sequence **predicted molecular weight based on aminoacid sequence choosed for cloning (without tags or signal sequences)

Supplementary Table S3: Performance of anti-Spike antibodies in different techniques on variant Spike/RBD proteins or variant SARS-CoV-2 infected cells

Method	ELISA				Flow cytometry			IF
**Variant**	B.1.617	B.1.351	B.1.617.2	B.1.1.529*	B.1.1.7	B.1.351	B.1.617.2	B.1.617.2
RBD.01	+	+	+	+	+	+	+	ND
RBD.03	+	-	+	-	ND	ND	ND	+
RBD.05	-	-	-	-	ND	ND	ND	ND
RBD.07	-	-	-	-	ND	ND	ND	ND
S1.01	+	+	+	-	+	+	+	+
S1.05	+	+	+	-	ND	ND	ND	+
S1-S2.14	+	+	+	-	ND	ND	ND	ND

S1-S2.15	ND	ND	ND	ND	ND	ND	ND	ND
S1-S2.16	+	+	+	-	ND	ND	ND	ND
S1-S2.17	ND	ND	ND	ND	ND	ND	ND	ND
S1-S2.18	+	+	+	-	ND	ND	ND	ND
S1-S2.19	ND	ND	ND	ND	ND	ND	ND	ND
S1-S2.20	+	+	+	-	ND	ND	ND	ND
S1-S2.21	ND	ND	ND	ND	ND	ND	ND	ND
S1-S2.22	+	+	+	-	ND	ND	ND	-

* mutated RBD protein, other variants used in ELISA are full Spike protein
